# A nomogram to predict postoperative surgical site infection of adult patients who received orthopaedic surgery: a retrospective study

**DOI:** 10.1038/s41598-023-34926-x

**Published:** 2023-05-19

**Authors:** Xu’an Huang, Yang Guo, Ribin Fu, Hongwei Li

**Affiliations:** 1 Department of Orthopedics, Peking Union Medical College Hospital, Chinese Academy of Medical Sciences and Peking Union Medical College, Graduate School of Peking Union Medical College, Beijing, People’s Republic of China; 2 The School of Clinical Medicine, Fujian Medical University, The First Affiliated Hospital of Xiamen University, 55 Zhenhai Road, Xiamen, 361001 Fujian Province People’s Republic of China; 3 The School of Clinical Medicine, Fujian Medical University, Zhongshan Hospital Xiamen University, No.201-209, Hubinnan Road, Siming District, Xiamen, 361001 Fujian Province People’s Republic of China; 4 Zhongshan Hospital Xiamen University, Xiamen, China

**Keywords:** Diseases, Health occupations, Medical research, Risk factors

## Abstract

Surgical site infection is a common postoperative complication with serious consequences. This study developed a nomogram to estimate the probability of postoperative surgical site infection for orthopaedic patients. Adult patients following orthopaedic surgery during hospitalization were included in this study. We used univariate and multivariate logistic regression analyses to establish the predictive model, which was also visualized by nomogram. To evaluate the model performance, we applied the receiver operating characteristic curve, calibration curve, and decision curve analysis, which were utilized in external validation and internal validation. From January 2021 to June 2022, a total of 787 patients were enrolled in this study. After statistical analysis, five variables were enrolled in the predictive model, including age, operation time, diabetes, WBC, and HGB. The mathematical formula has been established as follows: Logit (SSI) = − 6.301 + 1.104 * (Age) + 0.669 * (Operation time) + 2.009 * (Diabetes) + 1.520 * (WBC) − 1.119 * (HGB). The receiver Operating Characteristic curve, calibration curve, and decision curve analysis presented a good performance of this predictive model. Our nomogram showed great discriminative ability, calibration, and clinical practicability in the training set, external validation, and internal validation.

Surgical site infections (SSIs) are defined as infections that occur after surgery including superficial, deep, and organ-space^1^. Surgical site infection (SSI) happens in about 5% of patients following surgery, increasing the average hospital length of stay for more than 9 days, risk of mortality by up to 11-fold, and costs of hospitalization by no less than $20,000 per hospital stay^2^.

In the orthopaedic department, we often use internal fixation or other implant devices during surgery. On the one hand, these devices are foreign objects to the human body, which can be attacked by the autoimmune system. On the other hand, most of the internal fixation materials will stay in the body for a long period. These materials provide space and attachments for the reproduction of bacteria. Hence, the odds of infection not only exist in the early period after surgery but also exist in the future life of the patient. Once the SSI happens and the internal fixation gets infected, the patient should be treated with antibiotics for a long time. Furthermore, if the infection gets worse, the internal fixation should be removed immediately.

To deal with this situation, surgeons and researchers formulated many clinical guidelines^1–5^. These guidelines introduce SSIs systematically from many angles. However, they are hard to remember. According to McLaren et al^5^, anemia, obesity, HIV/AIDS, depression, dementia, immunosuppressive medications, and duration of hospital stay are factors that increase the risk of infection, some of which could be changed before surgical intervention. Thus, there is a demand to develop a clinical prediction model which can assist surgeons to foresee and alter the risk of SSIs.

Nomogram is a simple picture tool to predict clinical outcomes^6^. Through literature retrieval, we discovered that few studies had developed a nomogram for predicting the risk of SSIs in adult patients following orthopaedic surgery. Therefore, we aimed to investigate significant predictors that were associated with SSIs in adult patients following orthopaedic surgery. Also, we developed a nomogram to predict the risk of SSIs. Finally, we conducted external and internal validation to verify our prediction model.

## Results

### Correlation between risk factors and SSI

Through Fisher’s Exact Test and Pearson Chi-Square test, we summarized the correlation between possible risk factors and SSIs. Factors including age (*P* < 0.001), operation time (*P* < 0.001), operation history (*P* = 0.006), diabetes (*P* < 0.001), hypertension (*P* = 0.004), WBC (*P* < 0.001), HGB (*P* < 0.001), RBC (*P* = 0.013), FBG (*P* < 0.001), GLO (*P* = 0.014), P (*P* = 0.039) are statistically correlated to the happen of SSIs (Supplemental Digital Content 1).

### Univariate logistic regression analysis

According to Table 1, 9 variables are statistically correlated to SSIs (*P* < 0.05). For SSI, elderly patients (> 65 years old) have a higher odds ratio (OR) of 5.429 (95% confidence interval (CI), 2.255–13.069, *P* < 0.001) than younger patients (≤ 65 years old). The OR for SSI is 1.894 (95% CI, 1.240–2.895, *P* = 0.003) in patients with longer operation time. In patients with diabetes, the OR for SSI is 7.803 (95% CI, 3.991–15.255, *P* < 0.001). Also, patients who have high HGB have a lower OR of 0.263 (95% CI, 0.140–0.493, *P* < 0.001) for SSI than other patients. Apart from that, patients with high WBC have a higher OR of 4.778 (95% CI, 2.557–8.928, *P* < 0.001) for SSI than patients with normal or low WBC. Furthermore, the high occurrence of SSI is associated with operation history (OR, 2.585, 95% CI, 1.177–5.680, *P* = 0.018) and hypertension (OR, 2.093, 95% CI, 1.111–3.946, *P* = 0.022). Moreover, high level of FBG (OR, 2.235, 95% CI, 1.214–4.112, *P* = 0.010) and GLO (OR, 2.856, 95% CI, 1.493–5.465, *P* = 0.002) are also related to SSI (Table 1).

**Table 1:** Result of variables that are statistically significant in univariate logistic regression analysis.

Variables	*P*-value	OR	95%CI	
Age	0.000*	5.429	2.255	13.069
Operation time	0.003*	1.894	1.240	2.895
Operation history	0.018*	2.585	1.177	5.680
Diabetes	0.000*	7.803	3.991	15.255
Hypertension	0.022*	2.093	1.111	3.946
WBC	0.000*	4.778	2.557	8.928
HGB	0.000*	0.263	0.140	0.493
FBG	0.010*	2.235	1.214	4.112
GLO	0.002*	2.856	1.493	5.465

*OR* odds ratio, *95% CI* 95% confidence interval.

*P < 0.05.

### Correlations and collinearity between statistically significant variables

According to the result of collinear analysis, all variables have VIF < 3 and tolerance > 0.10, which statistically demonstrates that no collinearity exists between these variables (Supplemental Digital Content 2). According to the Spearman matrix of correlation coefficients, the absolute value of every correlation coefficient is less than 0.700, which statistically illustrates that these variables are not correlated to each other (Supplemental Digital Content 3).

### Multivariate logistic regression analysis

According to the result of the model comparison, only the model using enter method has higher AIC and BIC than the rest models (Supplemental Digital Content 4). Hence, we chose the model using the backward LR method to represent the five models with lower AIC and BIC as the optimal model since the model with the least AIC and BIC has the best imitative effect. Table 2 shows the result of multivariate logistic regression analysis using the backward LR method (Table 2). After multivariate logistic regression analysis, five out of nine variables remained statistically significant, which were age (OR, 3.017, CI, 1.121–8.125, *P* = 0.029), operation time (OR, 1.953, CI, 1.182–3.225, *P* = 0.009), diabetes (OR, 7.453, CI, 3.422–16.230, *P* < 0.001), WBC (OR, 4.573, CI, 2.257–9.266, *P* < 0.001), HGB (OR, 0.327, CI, 0.161–0.662, *P* = 0.002).

**Table 2:** Multivariate logistic regression analysis of predictors in the optimal model.

Variables	OR	95% CI	*P* value
Age	3.017	1.121, 8.125	0.029
Operation time	1.953	1.182, 3.225	0.009
Diabetes	7.453	3.422, 16.230	< 0.001
WBC	4.573	2.257, 9.266	< 0.001
HGB	0.327	0.161, 0.662	0.002

*WBC* white blood cell count, *HGB* hemoglobin, *OR* odds ratio, *CI* confidence interval.

### Mathematical formula and nomogram

**Figure 1:**
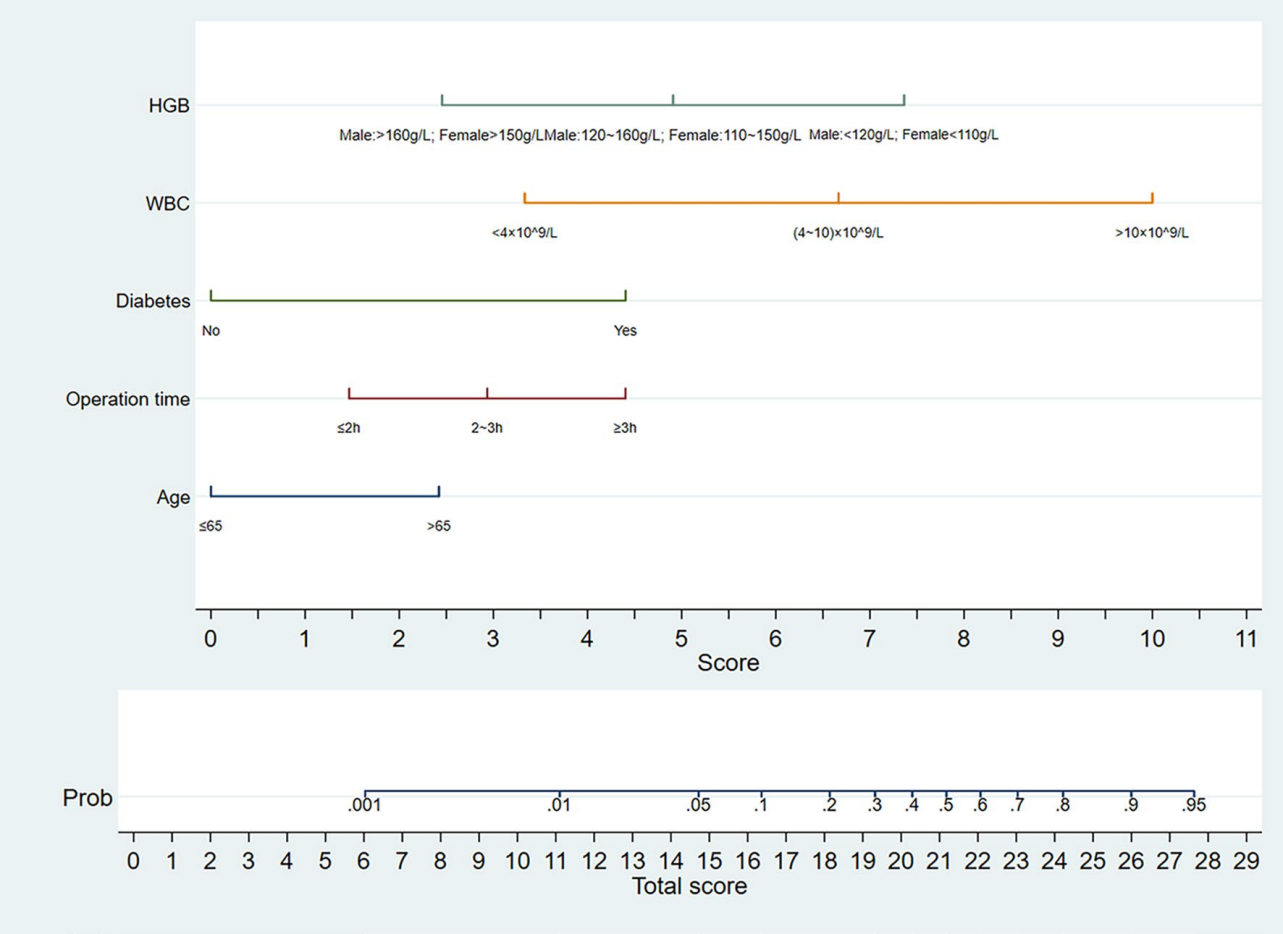
Nomogram for predicting the risk of SSI in patients. *HGB* Hemoglobin, *WBC* White blood cell count, *Prob* Probability.

Mathematical formula of our predictive model is as follows: Logit (SSI) = − 6.301 + 1.104 * (Age) + 0.669 * (Operation time) + 2.009 * (Diabetes) + 1.520 * (WBC) − 1.119 * (HGB). This predictive model was also visualized through a nomogram (Fig. 1).

### Performance of predictive model in the training set

**Figure 2:**
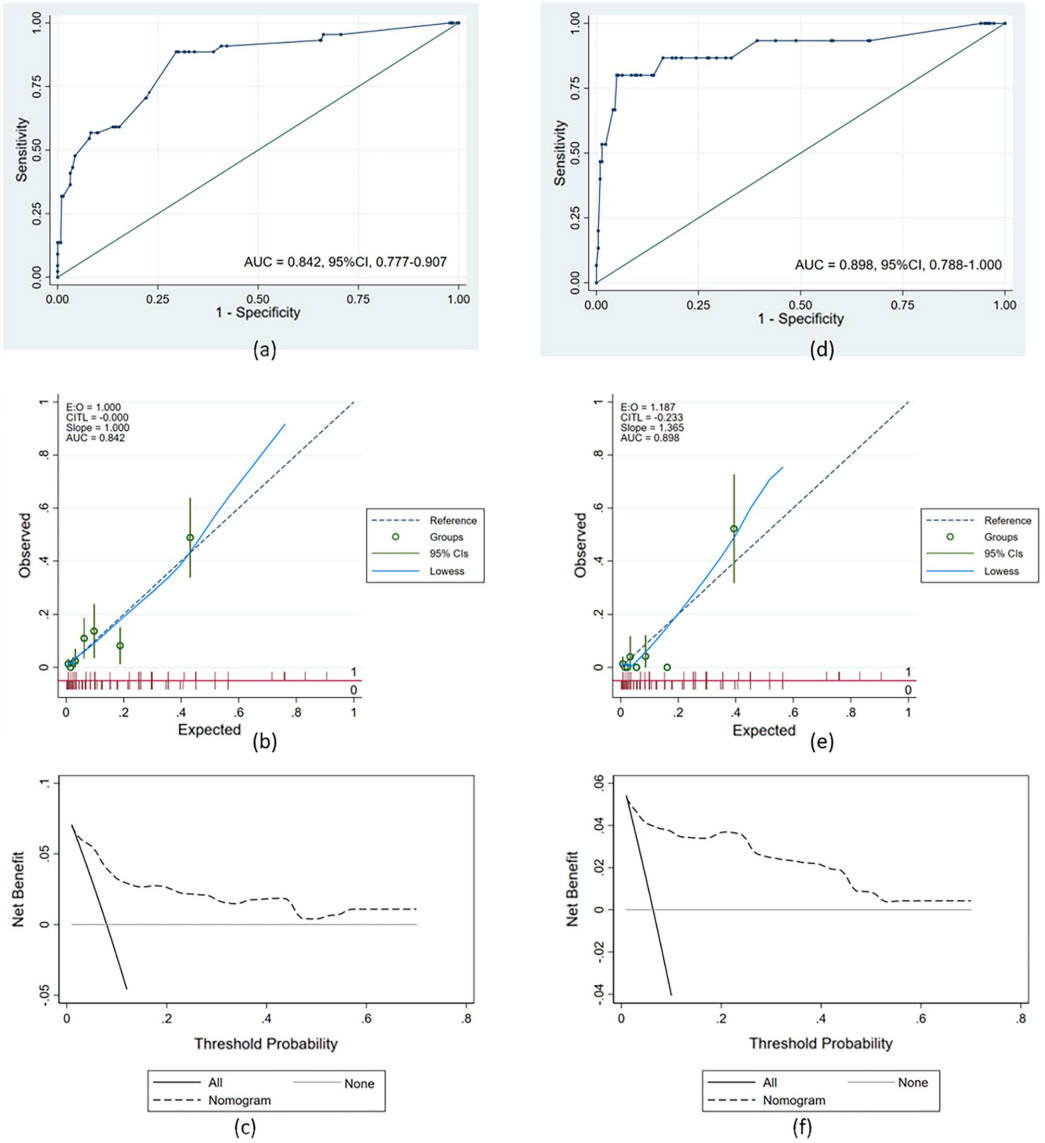
Performance of predictive model in training set and external validation set: The receiver operating characteristic curve (**a**), calibration curve (**b**), and decision curve analyses (**c**) of training set. The receiver operating characteristic curve (**d**), calibration curve (**e**), and decision curve analyses (**f**) of external validation set. *AUC* Area under curve, *CI* Confidence interval.

The performance of the predictive model was assessed through discriminative ability, calibration, and DCA in the training set. Figure 2a presents the area under ROC curve (AUC) is 0.842 (95%CI, 0.777–0.907) (Fig. 2a). Through the method of Youden’s index^13^, the cutoff point of this ROC curve is 0.068 (Sensitivity = 0.886, Specificity = 0.704). As for calibration, the calibration curve of the predictive model is highly close to the reference line (Slope = 1.000, CITL = -0.000) (Fig. 2b). Moreover, the result of the Hosmer–Lemeshow test (*P* = 0.299 > 0.05) in the training set reveals good calibration in this predictive model. Also, Fig. 2c presents the DCA curve in the training set. When the threshold probability is 0.1–0.7, using this predictive model can bring more benefits to patients (Fig. 2c).

### Performance of predictive model in the external validation set

The SSI rate in the external validation set is 6.4% (15/236). To compare with the training set (8.0%, 44/551), the Pearson Chi-Square test was used and the result (*P* = 0.464 > 0.05) shows there is no statistically significant difference in SSI rate between both sets. The performance of the predictive model was also evaluated through discriminative ability, calibration, and DCA in the external validation set. Figure 2d presents the area under ROC curve (AUC) is 0.898 (95%CI, 0.788–1.000) (Fig. 2d). Through the method of Youden’s index, the cutoff point of this ROC curve is 0.236 (Sensitivity = 0.800, Specificity = 0.950). Next, the calibration curve of the predictive model is close to the reference line (Slope = 1.365, CITL = −0.233), which shows our predictive model still has great calibration in the external validation set (Fig. 2e). Furthermore, the result of the Hosmer–Lemeshow test (*P* = 0.372 > 0.05) in the validation set discloses the nice calibration performance of this predictive model. In addition, our predictive model can bring more net benefit to patients in the external validation set, when the threshold probability is 0.1–0.7 (Fig. 2f).

### Repeatability and internal validation

**Figure 3:**
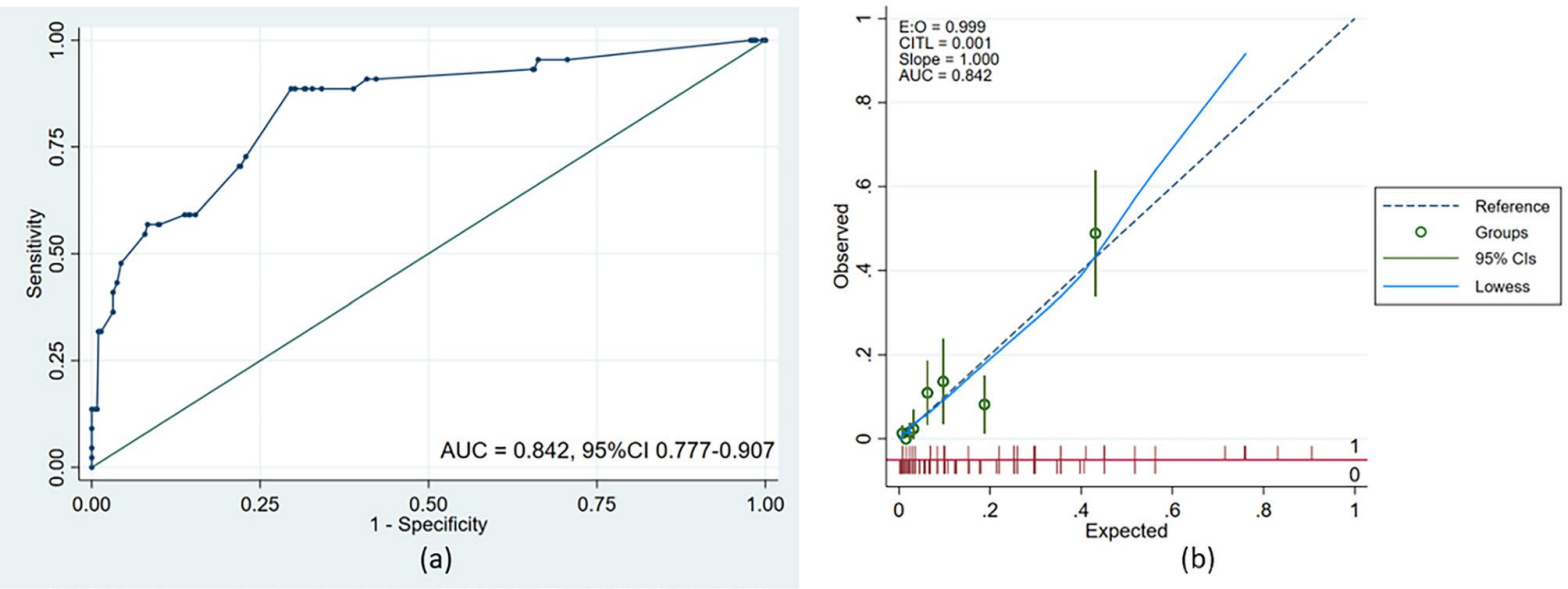
Repeatability and internal validation: The receiver operating characteristic curve (**a**) and calibration curve (**b**) of internal validation through bootstrap. *AUC* Area under curve, *CI* Confidence interval.

Our bootstrap results were based on 1000 bootstrap samples. The mathematical formula of the predictive model after bootstrap sampling is similar to the original one (Supplemental Digital Content 5). ROC curve (Fig. 3a) and calibration curve (Fig. 3b) have been drawn to estimate the predictive model after Bootstrap. The AUC of this model is 0.842 (95%CI, 0.777–0.907). Calibration curve of this model is highly close to the reference line (Slope = 1.000, CITL = 0.001).

## Discussion

In this study, we established a predictive model that could effectively estimate the probability of SSI for orthopaedic patients. Data of 551 patients (training set) and 236 patients (validation set) was analyzed. Depending on the diagnostic standard of SSI, both sets were divided^5^. Firstly, through Pearson Chi-Square Test and Fisher’s Exact Test, we can see age, operation time, operation history, diabetes, hypertension, WBC, HGB, RBC, FBG, GLO, P are potential risk factors for SSI. Secondly, after conducting logistic regression, the mathematical formula is as follows: Logit (SSI) = − 6.301 + 1.104 * (Age) + 0.669 * (Operation time) + 2.009 * (Diabetes) + 1.520 * (WBC) − 1.119 * (HGB). Next, the model was visualized through a nomogram. After that, the predictive model shows great discriminative ability, calibration, and much net benefit for patients in both the training set and external validation set^14^. Comparing with training set, the predictive model shows better discriminative ability, slightly worse calibration and more net benefit for patients in external validation set. Moreover, internal validation using bootstrap presents nice repeatability, which further demonstrates the good performance of the model.

Nomogram can be used as a routine tool to predict the probability of adult patients following orthopaedic surgeries, which can help doctors to make clinical decisions to prevent the early and later stage of infection. SSI criteria of the National Surgical Quality Improvement Program (NSQIP) and National Healthcare Safety Network (NHSN) are traditional tools to diagnose SSIs^15^. Compared with them, our nomogram is easier to use and more suitable for patients following orthopaedic surgeries. All the continuous variables were converted into categorical variables for easier corresponds to the scale and variables included in this nomogram are commonly used in daily clinical practice. Also, all variables are objective in our nomogram, which provides good stability for predicting outcomes.

There were some differences between our study and other studies concerning the clinical predictive model. First and foremost, many clinical predictive models were single-center and used only internal validation since the external validation set is harder to obtain than the internal validation set^16,17^. Otherwise, external and internal validation were both used in our predictive model, making our model more convincing. Moreover, our predictive model aims for all adult patients following orthopaedic surgeries. This was different from other studies that targeted patients with particular conditions, for instance, Peng et al^18^ formulate a nomogram for elderly patients with hip fracture, and Wang et al^19^ establish a model for nonfracture patients following total hip arthroplasty. From this point of view, our predictive model has wider clinical applicability than others. Furthermore, we had a bigger sample size of 787 patients than other studies, which can bring us a stabler model in the larger quantity of patients^20,21^. In addition, different from Ma et al^17^, laboratory tests in our study were performed after operation, taking the influence of operation into account may increase the accuracy of our model.

HGB (OR, 0.327, CI, 0.161–0.662, *P* = 0.002) acted as a protective factor in our predictive model. Undiagnosed anemia (Female: HGB < 110 g/L, Male: HGB < 120 g/L) is common in orthopaedic surgical patients^22^. It is one of the modifiable risk factors for SSI and correction of hemoglobin may reduce the likelihood of postoperative SSI^23^. Moreover, higher perioperative hemoglobin concentrations in patients with orthopaedic diseases are reported to be related to shorter length of hospital stay and lower mortality^24^. Also, it is convenient for doctors to monitor patients’ HGB by utilizing blood routine examinations. Therefore, controlling patients’ hemoglobin at a normal or even slightly higher level is needed to lower the rate of SSI. Once the HGB is lower than the normal level, a more hematological examination should be done to determine the type of anemia. For patients confirmed with iron deficiency anemia, iron supplementation is a must. The effectiveness of oral iron in the management of preoperative anemia has been demonstrated in patients undergoing orthopaedic surgery^25^. Apart from that, Goodnough et al^22^ suggest that erythropoiesis-stimulating agents (ESA) therapy should be used for anemic patients in whom nutritional deficiencies have been ruled out, corrected, or both.

WBC is a well-known laboratory index associated with infection. The postoperative WBC counts are significantly higher in the infected patients than in the noninfected patients on a postoperative day^26^. Increased WBC count postoperatively is a reliable laboratory index, which prompts doctors to assess the surgical wound more carefully. More precise diagnostic tools, such as contrast-enhanced CT, contrast-enhanced MRI, and PET-CT, can be used accordingly to achieve the final diagnosis^27^. Hence, daily monitoring of WBC count can help doctors to estimate the incidence of SSI for patients postoperatively, which is accessible to most medical institutions and affordable for patients.

With the rising morbidity of diabetes worldwide, the quantity of diabetic patients requiring orthopaedic surgeries is expected to increase in the future^28^. Furthermore, according to Martin et al^29^, a significant association between diabetes and SSIs is found across multiple types of surgeries. Even in the situation that perioperative hyperglycemia is controlled, a history of diabetes still acts as an independent risk factor for SSI. The occurrence of subsequent immune suppression and perioperative hyperglycemia is influenced by the complex contributions of factors in addition to the diabetic history of the patient, including external glucose administration and physiologic stressors^30^. Thus, diabetic history and hyperglycemia caused by diabetes are both risk factors for the incidence of SSI. To remove this risk factor, Cheuk et al^31^ used perioperative intravenous insulin infusions. As a result, although about 10% of patients developed superficial SSIs postoperatively, deep SSIs were uncommon. Thus, diabetes is an effective predictive factor for SSIs which is commonly used and also could be treated by hypoglycemic drugs to prevent SSIs.

It is shown in our nomogram that operation time is a significant risk factor for SSI (OR, 1.953, CI, 1.182–3.225, *P* = 0.009). Normally, operation time depends on the surgeon’s technic, problems encountered during the operation process, and also the patient’s body condition. Longer operation time will lead to more exposure to incision, which provides chances for pathogens to invade. Also, longer operation time may greatly increase the tiredness of surgeons and nurses, and the risk of infection caused by accident violation of asepsis may rise. Moreover, Kurmann et al^32^ point out that the association between operation time and the risk of SSI seems to be linear. So, we can see the importance of controlling operation time to prevent SSIs. And measures like better preoperative preparation and more rational surgical arrangements can be applied to shorten the duration of surgery.

Age (OR, 3.017, CI, 1.121–8.125, *P* = 0.029) is a strong predictor of SSI in our study. With the increase of age, the immune system and organ function of the human body get worse gradually. Thus, under the muti-pressure of surgical trauma and low immunity, SSIs are more likely to occur in elderly patients. According to our study, patients no less than 65 years old have approximately 3 times the probability of SSI than patients under 65 years old. Kaye et al^33^ draw a similar conclusion that age ≥ 65 years is associated with increased risk of SSI when age is studied as a dichotomous variable. From this angle, elderly patients need to be evaluated thoroughly before an operation, to make sure their health conditions can afford the trauma caused by the operation. Besides, this variable is readily available, objective, and routinely collected by most hospitals, which is merit for our predictive model.

However, our study has some limitations. First, this is a retrospective study, which indicates that selection bias is inevitable in this study. Second, some patients are not included in our study because of too many missing values, which decreases our sample size. Thirdly, some risk factors that may be related to SSIs like C-reactive protein (CRP), glycosylated hemoglobin (HbA1C), Erythrocyte Sedimentation Rate (ESR), and Procalcitonin (PCT) do not have enough records in our electronic medical record system. Hence a prospective observational study with a better data collection system can be done in the following study.

In summary, a combination of HGB, WBC, diabetes, operation time, age are significant predictors of SSIs in patients following orthopaedic surgeries. We converted this result into a nomogram to predict the probability of SSIs. Our predictive model shows great discriminative ability, calibration, and clinical practicability in the training set. Moreover, external and internal validation both obtained satisfactory results. Doctors can utilize this nomogram to estimate the risk of postoperative SSIs in adult patients following orthopaedic surgeries so that effective interventions can be made as soon as possible to lower the medical and financial burden for patients.

## Methods

### Patients and ethics

**Figure 4:**
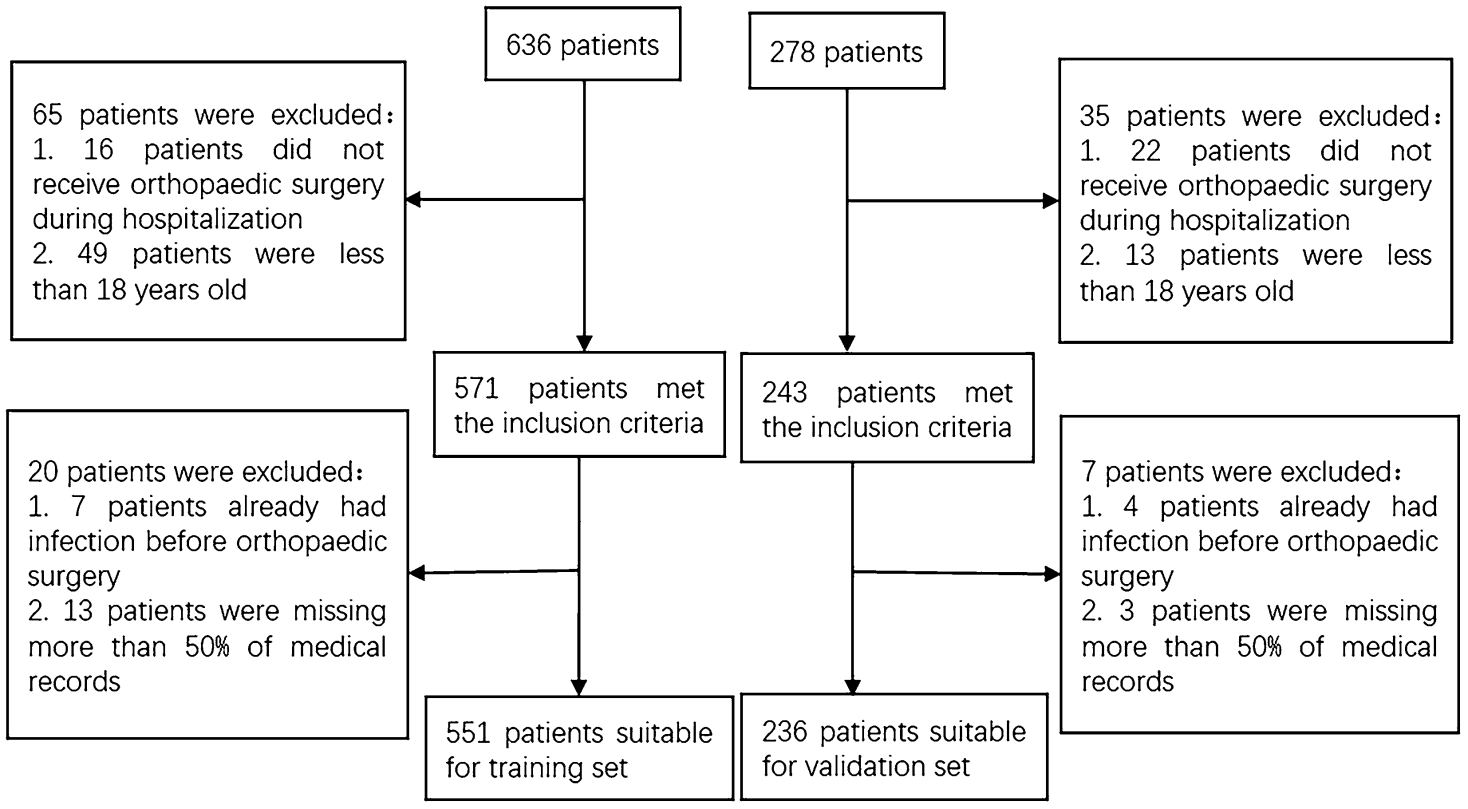
According to the inclusion and exclusion criteria, 551patients were enrolled in training set, 236 patients were enrolled in validation set.

A total of 787 patients following orthopaedic surgery were enrolled in this study according to inclusion and exclusion criteria. 551 patients from Zhongshan Hospital Xiamen University were enrolled in the training set. 236 patients from The First Affiliated Hospital of Xiamen University were enrolled in the validation set (Fig. 4). Number of patients corresponding to each classification of surgery included in this study was summarized (Supplemental Digital Content 6). 59 patients were diagnosed with SSI. We collected data from January 2021 to June 2022. Surgeries were performed by senior doctors in the orthopaedic department. To improve nutrition and hypoalbuminemia preoperatively, treatments like intravenous amino acids, oral amino acids, and albumin injection were conducted as nutritional supplementation. For all patients, antibiotics were used within 2 h of the skin incision. Antibiotics would be used for 3 days and then would be continued depending on the condition of the incision, body temperature, white blood cell count, etc. A venous pressure pump and low molecular weight heparin were conducted after the operation to prevent deep venous thrombosis^7^.

This study was approved by the Ethical Committees of Zhongshan Hospital Xiamen University and The First Affiliated Hospital of Xiamen University. All procedures of this study were conducted following the Declaration of Helsinki and informed consent was obtained from all patients.

### Inclusion and exclusion criteria

*Inclusion criteria*

1. Patients who received orthopaedic surgery during hospitalization.
2. Patients were no less than 18 years old.

*Exclusion criteria*

1. Patients already had the infection before orthopaedic surgery. The infection includes surgical site infection (SSI) and non-surgical site infection. For SSI, the diagnosis was based on the diagnosis standard below, and non-surgical site infection was diagnosed when one of these criteria were met: white blood cell count > 10*10^9/L, neutrophil ratio > 70%, lymphocyte ratio > 40%, procalcitonin > 0.5 µg/L, C-reactive protein > 2.87 mg/L.
2. Patients missing more than sixteen clinical and laboratory variables listed below.

### Diagnostic criteria

1. Patients without any records of infection preoperatively who had a diagnosis of SSI postoperatively were regarded as developing SSI.
2. The diagnosis of SSI was based on *Systematic Literature Review on the Management of Surgical Site Infections* (June 2018) published by the American Academy of Orthopaedic Surgeons (AAOS)^5^.

### Clinical and laboratory variables

All clinical and laboratory data were collected through electronic medical records. Variables that may be related to SSI were enrolled in our study, including sex, age, ABO blood type, systolic pressure, surgical wound classification (SWC), operation time, operation history, diabetes, hypertension, hyperlipidemia, heart disease, cerebrovascular disease, chronic lung disease, venous disease, white blood cell count (WBC), blood platelet count (PLT), hemoglobin (HGB), red blood cell count (RBC), mean corpuscular hemoglobin concentration (MCHC), red cell distribution width-variable coefficient (RDW-CV), hematocrit (HCT), fasting blood glucose (FBG), globulin (GLO), albumin (ALB), total protein (TP), prothrombin time (PT), activated partial thromboplastin time (APTT), serum phosphorus (P), serum calcium (Ca), total bilirubin (TBIL), alanine aminotransferase (ALT), asparagine aminotransferase (AST)^7–11^. All laboratory tests were performed 12 h after surgery.

### Statistical analysis

We used Excel to set up the original database. SPSS 26.0 (IBM Corp., Armonk, NY, USA) statistical software was used to analyze the data. When the number of missing values was greater than 10%, the variable would be removed. Otherwise, mean would be used to interpolate the missing value for continuous variables and mode would be used to interpolate the missing value for categorical variables. All the continuous variables were converted into categorical variables for a better presentation of the nomogram (Supplemental Digital Content 7). The association between clinical characteristics and SSI was performed using the Chi-Square test and Fisher’s Exact Test. If no more than 20% of cells had an expected count of less than 5, we would use the Pearson Chi-Square test. If more than 20% of cells had an expected count of less than 5 or the minimum expected count was less than 1, we would use Fisher’s Exact Test. When *P*-value < 0.05, the association was considered statistically significant.

The nomogram was established and verified with Stata 15 (Stata Corp., College Station, TX) software. First, we did univariate logistic regression to determine the potential variables for SSI. When *P*-value < 0.05, the corresponding variable would be considered statistically significant. Second, Spearman’s rank correlation coefficient was used to analyze the correlations between statistically significant variables. If correlation coefficients > 0.700 between different variables, the strongly correlated variables would be removed. To detect collinearity, the variance inflation factor (VIF) was calculated. If VIF > 3.000 or tolerance < 0.100, the corresponding variable would be removed. Next, we conducted a multivariate logistic regression analysis of all the statistically significant variables to examine their independence. Six different methods were used to do multivariate logistic regression analysis, including enter, backward LR, forward LR, backward stepwise, forward stepwise, forward, backward stepwise. Akaike Information Criterion (AIC) and Bayesian Information Criterion (BIC) of these models were calculated to choose the optimal model. Then, the predictive model would be visualized by nomogram. After that, we did external validation, including discriminative ability, calibration, and decision curve analysis (DCA). For discriminative ability, we used the receiver operating characteristic curve (ROC). For calibration, a calibration curve was established to measure how the probability evaluated by our model was close to the observed probability^12^. Also, the Hosmer–Lemeshow test was conducted to evaluate the calibration of the model. For DCA, DCA curves were drawn to assess the net benefit and optimal diagnostic threshold probability. Finally, the Bootstrap sampling method was used to verify the repeatability of the model.

## Supplementary Information

Supplementary Information.

## Data Availability

The datasets analysed in this study are not publicly available due to protecting patients’ privacy but are available from the corresponding author on reasonable request.
