# Supplementary material for: A nomogram to predict postoperative surgical site infection of adult patients who received orthopaedic surgery: a retrospective study
Source: Sci Rep. 2023 May 19;13:8129. doi: 10.1038/s41598-023-34926-x (PMC10199048; doi:10.1038/s41598-023-34926-x)
Supplement: Supplementary file 1 — Supplementary Information. [file 41598_2023_34926_MOESM1_ESM.pdf]

Supplementary information

A nomogram to predict postoperative surgical site infection of adult patients who received orthopaedic surgery: a retrospective study

Author names and final degree: Xu'an Huang, final degree: MBBS[1]

Yang Guo*, final degree: M.D.[2]

Ribin Fu*, final degree: MBBS[3]

Hongwei Li, final degree: M.D.[4]

The affiliation, and Address:

1. Department of Orthopedics, Peking Union Medical College Hospital, Chinese Academy of Medical Sciences & Peking Union Medical College, Graduate School of Peking Union Medical College, Beijing, China

2. The School of Clinical Medicine, Fujian Medical University, The First Affiliated Hospital of Xiamen University, China

3. The School of Clinical Medicine, Fujian Medical University, Zhongshan Hospital Xiamen University, China

4. Zhongshan Hospital Xiamen University, China

Co-corresponding Authors: Yang Guo, Ribin Fu

Name: Yang Guo

Email: g20041117@xmu.edu.cn

Phone: +86 0592-2139653

Fax: 0592-2139109

Address: 55 Zhenhai Road, Xiamen, Fujian Province, People's Republic of China, 361001

Name: Ribin Fu

Email: 13799786280@163.com

Phone: +86 0592-2993081

Fax: 0592-2993080

Address: No.201-209, Hubinnan Road, Siming District, Xiamen, Fujian Province, People's Republic of China, 361001

Legend of Supplementary Information

Supplemental Digital Content 1: Risk factors that are significantly correlated to SSI.

Supplemental Digital Content 2: Collinear analysis of characteristic variables

Supplemental Digital Content 3: Spearman matrix of correlation coefficients

Supplemental Digital Content 4: Comparison of multivariate logistic regression results

Supplemental Digital Content 5: Bootstrap result of variables in the equation

Supplemental Digital Content 6: Number of patients corresponding to each classification of surgery that included in this study

Supplemental Digital Content 7: Classification standard of variables

Supplemental Digital Content 1 Risk factors that are significantly correlated to SSI

**Risk factors**	**Total**	**SSI**		***P*-value**
	**(n=787)**	**No (n=728)**	**Yes (n=59)**	
Age				<0.001
≤65	411 (52.2%)	399 (97.1%)	12 (2.9%)	
>65	376 (47.8%)	329 (87.5%)	47 (12.5%)	
Operation time				<0.001
0-2h	611 (77.6%)	570 (93.3%)	41 (6.7%)	
2-3h	109 (13.8%)	105 (96.3%)	4 (3.7%)	
≥3h	67 (8.6%)	53 (79.1%)	14 (20.9%)	
Operation history				0.006
No	350 (44.5%)	334 (95.4%)	16 (4.6%)	
Yes	437 (55.5%)	394 (90.2%)	43 (9.8%)	
Diabetes				<0.001
No	704 (89.5%)	671 (95.3%)	33 (4.7%)	
Yes	83 (10.5%)	57 (68.7%)	26 (31.3%)	
Hypertension				0.004
No	603 (76.6%)	567 (94.0%)	36 (6.0%)	
Yes	184 (23.4%)	161 (87.5%)	23 (12.5%)	
WBC				<0.001
Low	18 (2.3%)	17 (94.4%)	1 (5.6%)	
Normal	580 (73.7%)	558 (96.2%)	22 (3.8%)	

High	189 (24.0%)	153 (81.0%)	36 (19.0%)	
HGB				<0.001
Low	174 (22.1%)	144 (82.8%)	30 (17.2%)	
Normal	597 (75.9%)	568 (95.1%)	29 (4.9%)	
High	16 (2.0%)	16 (100.0%)	0 (0.0%)	
RBC				0.013
Low	147 (18.7%)	128 (87.1%)	19 (12.9%)	
Normal	601 (76.4%)	565 (94.0%)	36 (6.0%)	
High	39 (4.9%)	35 (89.7%)	4 (10.3%)	
FBG				<0.001
Low	13 (1.7%)	12 (92.3%)	1 (7.7%)	
Normal	549 (69.8%)	525 (95.6%)	24 (4.4%)	
High	223 (28.3%)	190 (85.2%)	33 (14.8%)	
GLO				0.014
Low	16 (2.0%)	14 (87.5%)	2 (12.5%)	
Normal	462 (58.7%)	438 (94.8%)	24 (5.2%)	
High	309 (39.3%)	276 (89.3%)	33 (10.7%)	
P				0.039
Low	164 (20.8%)	150 (91.5%)	14 (8.5%)	
Normal	605 (76.9%)	564 (93.2%)	41 (6.8%)	
High	18 (2.3%)	14 (77.8%)	4 (22.2%)	

All continuous variables were converted to categorical variables. If no more than 20%

cells had expected count less than 5, Pearson Chi-Square test was used. If more than 20% cells had expected count less than 5 or the minimum expected count is less than 1, Fisher's Exact Test was used. When P-value<0.05, the correlation would be considered statistically significant.

Supplemental Digital Content 2: Collinear analysis of characteristic variables

Variables	Tolerance	VIF
Age	0.730	1.370
Operation time	0.966	1.035
Operation history	0.932	1.073
Diabetes	0.836	1.195
Hypertension	0.808	1.238
WBC	0.940	1.064
HGB	0.929	1.077
FBG	0.883	1.133
GLO	0.890	1.124

Supplemental Digital Content 3 Spearman matrix of correlation coefficients

Variables	Age	Operation time	Operation history	Diabetes	Hypertension	WBC	HGB	FBG	GLO
Age	1.000								
Operation time	-0.090	1.000							

Operation history	0.207	-0.108	1.000						
Diabetes	0.216	0.020	0.171	1.000					
Hypertension	0.364	-0.005	0.108	0.274	1.000				
WBC	0.140	0.074	0.003	0.009	0.150	1.000			
HGB	-0.246	-0.011	-0.094	-0.081	-0.046	-0.069	1.000		
FBG	0.185	0.089	0.041	0.281	0.173	0.130	-0.039	1.000	
GLO	0.284	0.005	0.039	0.154	0.199	0.151	-0.030	0.137	1.000

Supplemental Digital Content 4: Comparison of multivariate logistic regression results

Methods	df	AIC	BIC
Enter	10	243.095	286.212
Forward stepwise	6	238.451	264.321
Backward stepwise	6	238.451	264.321
Forward & Backward stepwise	6	238.451	264.321
Forward LR	6	238.451	264.321
Backward LR	6	238.451	264.321

AIC, Akaike's information criterion; BIC, Bayesian information criterion; df, degree of freedom.

Supplemental Digital Content 5: Bootstrap result of variables in the equation

	B	Bias	Std. Error	*P*-value	95% Confidence Interval	
Age	1.104	0.089	0.505	0.003	0.321	2.357
Operation time	0.669	0.010	0.296	0.014	0.067	1.263
Diabetes	2.009	0.050	0.414	0.001	1.258	2.968
WBC	1.520	0.024	0.430	0.001	0.733	2.413
HGB	-1.119	-0.015	0.367	0.002	-1.886	-0.461
Constant	-6.301	-0.196	1.512	0.001	-9.735	-3.706

Bootstrap results are based on 1000 bootstrap samples, WBC: White blood cell count, HGB: Hemoglobin.

Supplemental Digital Content 6: Number of patients corresponding to each classification of surgery that included in this study

Classification of surgery	Training set (patients)	Validation set (patients)
Internal fixation	289	97
External fixation	5	1
Removal of Internal fixation	31	13
Joint replacement	65	25
Revision of the joint	4	10
Excision of lesions	27	15
Tendon repair	13	9
Vertebralplasty	54	24
Suture of the meniscus	8	13
Release of tendon sheath	6	10
Cervical decompression and fusion	7	2
Lumbar discectomy	10	3
Correction of hallux valgus deformity	5	4
Arthroscopy	13	2
Amputation	4	5
Arthrolysis	10	3
Total	551	236

Supplemental Digital Content 7 Classification standard of variables

Variables	Classification standard
SSI	No
	Yes
Sex	Male
	Female
Age	≤65 years
	>65 years
ABO blood type	O
	A
	B
	AB
Systolic pressure	Low < 90 mmHg
	Normal 90-140 mmHg
	High >140 mmHg
SWC	I

	II
	III
	IV
Operation time	Low < 2h
	Normal 2-3h
	High > 3h
Operation history	No
	Yes
Diabetes	No
	Yes
Hypertension	No
	Yes
Hyperlipidemia	No
	Yes
Heart disease	No
	Yes

Cerebrovascular disease	No	
	Yes	
Chronic lung disease	No	
	Yes	
Venous disease	No	
	Yes	
WBC	Low < 4×10^9/L	
	Normal (4-10)×10^9/L	
	High > 10×10^9/L	
PLT	Low < 100×10^9/L	
	Normal (100-300)×10^9/L	
	High > 300×10^9/L	
HGB	Female	Male
	Low < 110 g/L	Low < 120 g/L
	Normal (110-150) g/L	Normal (120-160) g/L
	High > 150 g/L	High > 160 g/L

RBC	Female	Male
	Low < 3.5 ×10^12/L	Low < 4.0 ×10^12/L
	Normal (3.5-5.0) ×10^12/L	Normal (4.0-5.5) ×10^12/L
	High > 5.0 ×10^12/L	High > 5.5 ×10^12/L
MCHC	Low < 320 g/L	
	Normal (320-360) g/L	
	High > 360 g/L	
RDW_CV	Low < 11.5 %	
	Normal (11.5-14.5) %	
	High > 14.5 %	
HCT	Female	Male
	Low < 0.37 L/L	Low < 0.40 L/L
	Normal (0.37-0.48) L/L	Normal (0.40-0.50) L/L
	High > 0.48 L/L	High > 0.50 L/L
FBG	Low < 3.9 mmol/L	
	Normal (3.9-6.1) mmol/L	

	High > 6.1 mmol/L
GLO	Low < 20 g/L
	Normal (20-30) g/L
	High > 30 g/L
ALB	Low < 40 g/L
	Normal (40-55) g/L
	High > 55 g/L
TP	Low < 60 g/L
	Normal (60-80) g/L
	High > 80 g/L
PT	Low < 10 s
	Normal (10-16) s
	High > 16 s
APTT	Low < 26 s
	Normal (26-46) s
	High > 46 s

P	Low < 0.97 mmol/L<br>Normal (0.97-1.61) mmol/L<br>High > 1.61 mmol/L
Ca	Low < 2.25 mmol/L<br>Normal (2.25-2.58) mmol/L<br>High > 2.58 mmol/L
TBIL	Low < 3.4 μmol/L<br>Normal (3.4-17.1) μmol/L<br>High > 17.1 μmol/L
ALT	Low < 5 U/L<br>Normal (5-40) U/L<br>High > 40 U/L
AST	Low < 8 U/L<br>Normal (8-40) U/L<br>High > 40 U/L
